# The role of social media and infographics in increasing cultural heritage awareness of young athletes: an experimental study

**DOI:** 10.3389/fpsyg.2025.1687803

**Published:** 2025-10-09

**Authors:** Fikret Bademci, Hicran Hanım Halaç

**Affiliations:** ^1^School of Architecture, Faculty of Humanities and Social Sciences, University of Liverpool, Liverpool, United Kingdom; ^2^Department of Architecture, Faculty of Architecture and Design, Eskisehir Technical University, Eskisehir, Türkiye

**Keywords:** cultural heritage, sport psychology, social media, infographic, young athletes, awareness, digital education, identity

## Abstract

**Introduction:**

Preserving cultural heritage and passing it on to future generations strengthens societies’ ties to their past, increases a sense of belonging, and promotes individual and societal psychological well-being. The potential role of young people, especially athletes, in cultural heritage promotion and the increasing influence of digital tools in this process offer new opportunities for the dissemination of cultural awareness in the modern age.

**Objective:**

This study aims to experimentally investigate the effectiveness of a social media and infographic-based activity in the process of increasing the cultural heritage awareness levels of young athletes studying in official sports high schools affiliated to the Turkish Ministry of National Education.

**Methods:**

The study was conducted with 210 young athletes using a one-group pre-test/post-test design. After the pre-test was administered to the participants, infographics introducing Turkey’s cultural heritage included in the UNESCO World Heritage List were shared on a specially created social media account (Instagram) and the post-test was administered after a specified period of time. The quantitative data obtained were analyzed using frequency, percentage, dependent sample *t*-test and one-way ANOVA tests using IBM SPSS STATISTICS 22.0 software.

**Findings:**

As a result of the study, it was found that the implemented activity showed a statistically significant increase in the cultural heritage knowledge and awareness levels of young athletes from pre-test to post-test. There was no significant difference between the participants’ sports branches, regions of residence and grades on cultural heritage awareness in general; however, significant regional and grade differences were observed in some specific cultural heritage areas.

**Conclusion and recommendations:**

The findings suggest that social media and infographics are a highly effective and accessible tool for raising cultural heritage awareness among young athletes. This methodology offers an innovative and psychologically effective approach to the preservation and transmission of cultural heritage to future generations, while contributing to the strengthening of young people’s cultural identity and sense of social belonging. For future studies, research that examines the psychosocial effects of digital tools in more depth and designs long-term awareness projects is recommended.

## 1 Introduction

The development of young athletes should be addressed with a holistic approach that goes beyond just physical abilities and sporting performance but also encompasses psychosocial and emotional dimensions. Sport participation offers a rich environment for young people to develop important life skills such as self-confidence, resilience, stress management, and social skills, as well as maintain physical health. This development enables young people to be successful in their lives outside of sport and in their overall well-being (Gary and Rubin, 2016; Yapar et al., 2025). Sport acts as a powerful catalyst, providing opportunities for young people to cope with challenges, overcome setbacks, and develop a strong sense of self-efficacy (Sinha, 2024).

The impact of emotional intelligence (EI) in the field of sport psychology is increasing. Emotional intelligence is defined as the ability to perceive, understand, manage, and express one’s own emotions and the emotions of others (Liao et al., 2021). In the context of sport, this skill directly affects athletes’ ability to make decisions under competitive pressure, cope with stress, maintain motivation, and communicate effectively with teammates (Gary and Rubin, 2016).

Preserving cultural heritage and passing it on to future generations strengthens societies’ ties to their past, enhances individual and societal sense of belonging, and promotes individual and societal psychological well-being. International organizations such as UNESCO emphasize the preservation and transmission of cultural heritage to future generations and its critical importance for youth identity formation and social integration (UNESCO, 2013). Sport itself is a universal cultural element and plays an important role in shaping young people’s cultural identity and social interactions (Sinha, 2024). Cultural identity is recognized as one of the cultural factors that profoundly influence athletes’ motivation, stress coping mechanisms, and overall well-being (Lee, 2025).

In the modern era, social media and digital tools such as infographics offer new opportunities to disseminate young people’s cultural awareness. By visually simplifying complex information and making it appealing, infographics not only capture young people’s attention but also allow them to process information with less mental effort. Given the social media usage habits of young athletes, these platforms maximize the accessibility and psychological engagement of cultural awareness interventions.

This study aims to link the findings of a social media and infographic-based experimental intervention to increase young athletes’ cultural heritage awareness around the themes of emotional intelligence, performance, coaching, and well-being. In this context, it will address gaps in the existing literature by examining potential direct and indirect links between cultural heritage awareness and young athletes’ emotional intelligence and well-being and provide a conceptual framework for future research and practice.

### 1.1 Purpose of the study and its contribution to the field of psychology

The main purpose of this study is to inform young athletes about cultural heritage and to instill cultural heritage awareness in them. In this context, the study aims to present an activity methodology based on social media and infographics in the process of increasing the awareness of young athletes about cultural heritage. The study aims to increase the awareness of young athletes about cultural heritage, to provide a better understanding of cultures among young people, to encourage young people to get involved in the protection of cultural heritage at local, national, and global levels, to develop intercultural awareness and competencies for athletes, to increase access to cultural heritage through digital tools, and to support the social development of young athletes as well as their skills.

By emphasizing the intersection of cultural competence and social responsibility in the field of sport psychology, this study reveals the potential of sport psychology to contribute to the psychosocial development of athletes not only as performance-oriented but also as cultural ambassadors. The importance of cultural competence for sport psychology professionals is emphasized. Increasing athletes’ awareness of cultural heritage enhances their overall cultural competence, thus demonstrating that the field of sport psychology should focus not only on athletic performance but also on athletes’ individual and social well-being. This offers a new perspective on the multidimensional development of athletes.

### 1.2 Research questions

In this direction, the main question of the research was determined as “Does an activity with an infographic design that can be applied on social media affect the cultural heritage awareness of athletes?”

The sub-questions of the research are as follows:

- Is there a significant difference between the pre-test and post-test results of sports high school students who participated in the cultural heritage promotion activity on social media with infographics?
- Is there a significant difference between the results of the cultural heritage promotion activity conducted through social media with infographics for sports high school students and the participants’ participation in team sports or individual sports?
- Is there a significant difference between the results of the cultural heritage promotion activity conducted for sports high school students through social media with infographics and the region where the participants live?
- Is there a significant difference between the results of the cultural heritage promotion activity carried out for sports high school students through social media with infographics and the grade of the participants?

## 2 Conceptual framework and literature review

### 2.1 Emotional intelligence and psychosocial development of young athletes

Emotional intelligence (EI) is a critical skill set that includes the ability to perceive, understand, manage, and express one’s own emotions and the emotions of others (Liao et al., 2021). These abilities are generally considered in four main dimensions: assessing and expressing one’s own emotions, assessing and recognizing others’ emotions, facilitating emotional thinking, and regulating emotions (Liao et al., 2021). In the context of sport, these skills directly affect athletes’ ability to cope with the stress they face in competitive environments, maintain motivation, communicate effectively with teammates, and make good decisions under pressure (Gary and Rubin, 2016).

Emotional intelligence development cannot be considered independent of the cultural context. While emotional intelligence involves an individual’s ability to manage and express emotions (Liao et al., 2021), culture profoundly influences how individuals express and regulate their emotions (Ford and Mauss, 2015). For example, some cultures promote emotional restraint (e.g., many Asian cultures), while others embrace more open expression (e.g., Western cultures). These cultural norms and expectations shape a young person’s development of emotional intelligence and which emotional regulation strategies to use in particular situations. It has been suggested that emotion regulation that is congruent with cultural values may be more adaptive (Ford and Mauss, 2015). In this case, increasing young athletes’ cultural heritage awareness may help them to better understand the norms of emotional expression and regulation in their own cultural context. This understanding may influence coping strategies to deal with stress and pressure encountered in the sport environment (Parnabas et al., 2018) and provide a basis for coaches to develop culturally responsive approaches (Lee, 2025). This link suggests that the development of emotional intelligence is not only an individual process but also a process of cultural learning and adaptation. Athletes’ understanding of their own cultural identity may allow them to evaluate their emotional reactions and the reactions of others from a broader cultural perspective, leading to a more in-depth and context-sensitive development of emotional intelligence. This allows athletes to not only learn to manage their emotions but also to align this management with their own cultural values.

### 2.2 Cultural heritage, identity, well-being, and the relationship between them

Cultural heritage is a concept that has many dimensions, such as movable, immovable, tangible, intangible, etc., and is widely used in many disciplines (Jagielska-Burduk et al., 2021). Cultural heritage is defined as a social process that is transmitted to future generations through traditions and artifacts and is continuously reconstructed with new contributions from future generations (Mason, 2006; Tait and While, 2009; While and Short, 2010; Avrami et al., 2000; Vakhitova, 2014; 218). The Council of Europe defines cultural heritage as a set of resources inherited from the past, which individuals define as a reflection of their lives and which are formed by the interaction of people and space over time (Council of Europe, 2005). The protection of cultural heritage is recognized as a global responsibility in terms of both its historical and esthetic values as well as the continuity of its cultural meanings (Worthing and Bond, 2008).

In the protection of cultural heritage, not only are legal and administrative efforts not sufficient, but also the conscious participation and education of the society are seen as fundamental requirements (Jagielska-Burduk et al., 2021; İslamoğlu, 2018). Especially raising cultural heritage awareness among children and young people at an early age is important for the establishment of a sustainable conservation approach in the future. With the World Heritage Education program launched in 1994, UNESCO aimed to involve young people in the protection of heritage and developed various materials and activities in this context.

The use of well-known individuals, especially athletes, as cultural ambassadors to raise awareness of cultural heritage in society is an important strategy because athletes can have a positive impact on youth and children through their trustworthy and influential roles (Milanovic et al., 2015). As seen in advertising, athletes are preferred as brand faces because they can appeal to large audiences and can be more effective than other celebrity groups in creating brand awareness and positive attitudes (Keat, 2015). The social impact of sport is felt in many areas, from the personality development of individuals to the welfare level of society, and in this respect, sport is considered as a powerful tool that can be used in solving social problems (İmamoğlu, 1992; Yetim, 2000; Milanovic et al., 2015). Sociology of sport, on the other hand, examines the social structure and effects of sport and puts this interaction on a scientific basis (Yetim, 2000). In this context, it is important for individuals to develop social belonging by recognizing their culture and to have cultural heritage awareness, especially at the beginning of their youth.

Cultural heritage is a key resource for young people to construct their identities, connect with their past, and develop a sense of social belonging. These connections support individual and social psychological well-being. Sport itself is a universal cultural element and plays an important role in shaping young people’s cultural identities and social interactions (Sinha, 2024). Especially for diasporic communities, sport participation functions as a means of reinforcing cultural identity, maintaining language and cultural practices, and creating a sense of belonging (Shin et al., 2025). Traditional sports and games also play an important role in preserving cultural heritage and empowering youth (UNESCO).

As a universal language, sport has the potential to bring together athletes from different backgrounds (Gary and Rubin, 2016). Team sports encourage social interaction and develop life skills such as cooperation, conflict resolution, and empathy (Sinha, 2024). Sport can also help overcome cultural differences, break down prejudices, and promote dialog (Council of Europe, 2005). This shows that sport is not only a physical activity but also a means of cultural integration and social cohesion.

Cultural competence in sport psychology refers to the ability to work effectively with athletes from different cultural backgrounds (Applied Sport Psychology, n.d.). This requires coaches and sport psychologists to understand athletes’ motivation, stress, coping mechanisms, and communication styles in a cultural context (Lee, 2025). Cultural competence is essential for creating inclusive, respectful, and supportive sport environments, which in turn increases athletes’ self-confidence and sense of belonging (The Aspen Institute, 2019). Cultural competence implies a paradigm shift from seeing differences as problems to seeing them as assets and using diversity as an educational and developmental tool.

There is a circular relationship between cultural heritage awareness and positive youth development. Cultural heritage awareness strengthens young athletes’ cultural identity and sense of belonging. This process enables young people to develop a clearer understanding of ‘who they are’ and ‘where they belong’. A strong sense of cultural identity and belonging increases individual self-confidence, psychological resilience, and overall well-being (Sinha, 2024). This strengthens the individual’s internal resources, making them more resilient to external challenges. This psychological resilience and well-being contribute to young athletes performing better in the sport environment, coping with stress more effectively, and developing emotional intelligence skills (Gary and Rubin, 2016). Sport serves as a laboratory where these skills are practiced and reinforced. Achievements, positive experiences, and peer/coach support in a sport environment can reinforce young people’s positive attitudes toward their cultural heritage and encourage them to adopt the role of cultural ambassadors. This allows cultural heritage to become not only knowledge that is learned but also an element of identity that is lived and shared. This leads to higher cultural awareness and positive identity development, creating a continuous cycle of development. This cyclical interaction reveals that cultural heritage awareness not only increases the knowledge level of young athletes but also serves as a multidimensional psychosocial resource that supports their positive development inside and outside of sport.

### 2.3 Digital tools (social media and infographics) and educational interventions

Social media play a central role in young people’s lives and provide a powerful platform for information access, communication, and interaction. Today, social media platforms have become an indispensable communication and identity-building tool for both institutional structures and individual actors in the field of sports. Institutions such as sports science faculties use Instagram as a professional tool to interact directly with their target audience of students and strengthen their institutional identity. Similarly, Olympic athletes also view this platform as a strategic medium for managing their personal brand image, advancing their careers, and connecting with their followers. This situation highlights the dual potential that social media offers for brand management at both the institutional and individual levels within the sports ecosystem (Yavuz and Semiz, 2022; Ertuğral et al., 2024). However, the use of social media, especially passive consumption, poses significant risks to the mental health of young athletes. Passive social media use has been shown to increase anxiety and decrease subjective well-being in young athletes, which is associated with upward social comparison (Zhang et al., 2023). This highlights the need for careful design of digital interactions and the importance of strategies to mitigate negative psychological effects.

Infographics are effective visualization tools that optimize the learning process by visually simplifying complex information and making it appealing. They facilitate the perception and retention of information and can strengthen critical thinking skills. Effective infographics condense information and support the development of additional meanings through visual clarity, composition, color, and visual reference points. Students’ positive perceptions of learning with infographics indicate that these tools offer a new learning environment and provide engaging, clear information (Bystrova, 2020).

The combination of social media and infographics offers an innovative and accessible methodology for cultural heritage promotion. The active involvement of the target audience (young people aged 14–18) in the selection of infographic characters reflects the principles of participatory design, making the content easier to adopt and more psychologically appealing to the target audience. This approach increases the level of belonging and interest of young people toward the content. Moreover, given the bidirectional effects of social media on young people’s identity formation and social interactions, promoting cultural heritage through these platforms acted as a psychological buffer that strengthened the positive aspects while reducing negative risks. This has the potential to increase proactive engagement and social responsibility awareness by encouraging young people’s transition from passive “digital consumers” to active “digital cultural ambassadors” of cultural heritage.

## 3 Methods

### 3.1 Research design

This paper has a multidisciplinary characteristic, covering the fields of architecture, sports, communication, and psychology. The focus of the study on cultural heritage necessitated the use of literature from the department of architecture, the scope of the study on young athletes necessitated the use of literature from the department of sports, the use of infographics and social media in the activity necessitated the use of literature from the department of communication, and the aim of raising cognitive awareness necessitated the use of literature from educational psychology.

In this study, quantitative research methods were utilized, and a one-group pre-test/post-test design, which is one of the weak experimental designs, was used. In this design, the researcher conducts a study on a single group and intervenes in the experimental group between the pre-test and post-test. Some reasons were effective in the pre-test-post-test design: it is not possible to control external variables in research such as education, promotion, and awareness raising; the fact that the research will be conducted in a certain process and while an intervention is made to the experimental group in this process, it is not known whether the participants in the control group are working on the subject or not; it is not ethically correct to create differences between students by creating experimental and control groups among students in the same schools and classes. Creswell (2012) stated that it is inherent in the nature of the research to use a one-group experimental design in the development and implementation of a new module. The choice of a one-group pre-test/post-test design for ethical and practical reasons reflects the validity-reliability dilemma often encountered in field studies, especially in social and educational psychology. This methodological choice demonstrates an attempt to balance applicability in field conditions and ethical responsibility for the welfare of participants, compromising internal validity. This acknowledges the methodological limitations of the study, but emphasizes its applicability and ethical sensitivity in a real-world context.

The one-group pre-test/post-test experimental design can be summarized as follows in Table 1.

**Table 1:** One group pre-test/post-test experimental design.

Group	Pre-test	Activity	Post-test
Participants	Demographic questions Cultural heritage achievement test	Sharing infographics prepared for participants to recognize cultural heritage on social media	Demographic questions Cultural heritage achievement test

In the one-group pre-test/post-test experimental design applied in this study, the dependent variables were determined as the participant’s sport branch, the region where the participant lived, and the participant’s grade. The independent variable, which is thought to have an effect on the dependent variable, is the infographic studies prepared for the promotion of cultural heritages included in the UNESCO World Heritage List within the borders of Turkey to be shared on social media.

### 3.2 Research group

In the study, the criterion sampling method, which is one of the purposeful sampling types, was used to determine the study group. The criterion was to be a student enrolled in official sports high schools affiliated with the Turkish Ministry of National Education. The research was applied to a total of 210 sports high school students in the 10th, 11th, and 12th grades studying in sports high schools across Turkey. The sample of 210 students consisted of 30 students from 7 geographical regions of Turkey. When the grades of the participants are analyzed, it is seen that 70 participants were 10th grade students, 70 participants were 11th grade students, and 70 participants were 12th grade students. For the consistency of the research data, in addition to selecting an equal number of students from each region and grade group, it was also ensured that the sports branches of the students (team sports or individual sports) were close in number. The selection of official sports high schools affiliated with the Ministry of National Education for the implementation of the study was influenced by the tendency of the students in these high schools toward sports, the fact that most of them are licensed in different sports branches, the increase in branch diversity, and the diversification of the age group. This age range (14–18 years) coincides with adolescence, a period of identity formation and intensive use of digital media.

### 3.3 Data collection tools

Two data collection tools were used in the study: pre-test and post-test. Both tests used in the research include the same questions. The tests consist of 6 questions to be used to determine the demographic characteristics of the participants and 18 questions to be used to determine whether the participants know cultural heritage. The section on cultural heritage consists of closed-ended multiple-choice questions prepared from the information in the infographics. Each question has 4 options. In the tests, demographic questions were asked to each participant in the same order, while questions about cultural heritage and the options of the questions were mixed. Preparing the cultural heritage questions directly from the infographic content provides strong content validity to directly and specifically measure the effectiveness of the intervention. This allows the observed increase in knowledge to be directly linked to the content presented. It means that the test is indeed measuring the knowledge presented during the intervention. If the questions had been more general, it might have been difficult to directly attribute the increase in knowledge to the infographics. This direct link increases the reliability of inferences about the effect of the intervention on cognitive outcomes.

### 3.4 Data collection

The link to the pre-test, which was transferred to the online form, was shared in the class social media groups of the students through the administrators. Students who completed the pre-test were asked to follow the Instagram account of the study, and those who did not were excluded from the study. The prepared infographics were shared as both posts and stories on the Instagram account named COSPORTS_TURKEY. The posts were shared three times a day (at 06:00, 12:00, and 19:00), as participants were found to use their social media accounts during these parts of the day. Data collection and content distribution based on social media usage habits (popular platform selection and strategic sharing times) maximized the accessibility and psychological engagement of the intervention in terms of integrating young athletes into their “digital habitat.” This increased the likelihood of the intervention reaching and influencing the target audience. The choice of Instagram and posting at specific times demonstrates the researchers’ understanding of the target audience’s digital behaviors. These strategic choices increased the likelihood that the content would be seen and engaged with by young athletes. This is not only a technical detail but also a practical application of psychological principles such as user experience and attention management, which contributes to the effectiveness of the intervention.

During the data collection process, 23 students who did not follow the Instagram account out of 338 students who participated in the pre-test were excluded from the study. When the application started, there were 315 students following the Instagram account. During the implementation, 24 students unfollowed the account, and these students were also excluded from the study. When the application was completed, the number of students following the Instagram account was 281. It was determined that 12 of these students did not complete the post-test and these students were excluded from the study. Twelve of the 269 students who participated in the post-test were excluded from the evaluation as a result of the reliability study. Of the remaining 257 students, 47 were excluded from the study in order to ensure consistency between the data, taking into account equal numbers in the variables of sport branch, region, and grade. Thus, the total number of people included in the evaluation was 210.

### 3.5 Data analysis and interpretation

The data from the pre-tests and post-tests administered to the volunteer participants were coded between 1 and 4 according to the number of options in the question and transferred to the IBM SPSS STATISTICS 22 ready-made statistical program. The data from the program were analyzed and interpreted in IBM SPSS STATISTICS 22 software according to frequency and percentage values. Thus, achievement scores were calculated on a question basis, and it was checked whether there was a statistically significant difference between the pre-test and post-test results.

When the normality distributions of the data were analyzed by Kolmogorov–Smirnov and Shapiro–Wilk analyses, it was concluded that the data did not show normal distribution, but it was assumed that the data showed normal distribution due to the sufficient number of samples according to the central limit theorem. Statistical literature suggests that according to the Central Limit Theorem, when the sample size exceeds 40 (*n* = 210 in this study), it is accepted that the distribution of sample means converges to normal and that parametric tests (*t*-test, ANOVA) are quite robust against such deviations. Therefore, the use of parametric tests in the analysis of the data was considered methodologically appropriate. As a result, the dependent sample *t*-test was applied. The dependent sample *t*-test was used to determine whether there was a significant difference between the pre-test and post-test results. The independent sample *t*-test was used to determine whether there was a significant difference between the results and the participants’ branches (team sport or individual sport). The ANOVA test was used to see whether the results showed a significant difference according to the participants’ grades and regions of residence. When it was determined that there was a significant difference in the ANOVA test results, the source of the differences was determined by looking at the equality of variance and applying Bonferroni tests when the variance was equal and Games-Howell tests when the variance was not equal.

## 4 Findings

In this section, the findings obtained from the analysis of the participants’ answers to the questions in the pre-test and post-test are presented. The answers given by the participants were analyzed by cross-referencing the information about the sport branch, the region they live in and the grade they are in.

The first sub-question investigated whether there was a significant difference between the pre-test and post-test results of sports high school students who participated in the cultural heritage promotion activity on social media with infographics. When the dependent sample *t*-test data in Table 2 are examined (*t*(209) = −18.208, *p* = 0.000 < 0.05), it is seen that there is a statistically significant difference between the pre-test and the post-test. This shows that the intervention provided to the participants through the activity enabled the participants to give more correct answers in the post-test. It was determined that the participants gained knowledge about cultural heritage through the activity.

**Table 2:** Dependent sample *t*-test results of the participants’ answers to the questions in the achievement test.

Test	*N*	X	S	*t*	SD	*p*
Pre-test	210	10, 25	0.285	−18.208	209	0.000
Post-test	210	14, 37	0.237			

Similar results were obtained for individual cultural heritage sites. For example, while 68.1% of the participants answered the question about “Great Mosque and Hospital of Divriği” correctly in the pre-test, this rate increased to 86.7% in the post-test. This increase was statistically significant (*t*(209) = −6.904, *p* = 0.000 < 0.05). The percentage of correct answers for “Historic Areas of Istanbul” increased from 70.5% in the pre-test to 90.0% in the post-test (*t*(208) = −7.125, *p* = 0.000 < 0.05). The percentage of correct answers for “Göreme National Park and the Rock Sites of Cappadocia” increased from 73.8% in the pre-test to 93.3% in the post-test (*t*(209) = −7.125, *p* = 0.000 < 0.05). For “Hattusha: the Hittite Capital,” the correct response rate increased from 45.2% in the pre-test to 75.7% in the post-test, for “Nemrut Dağ,” from 65.2 to 85.7%; and for “Hierapolis-Pamukkale,” from 68.6 to 82.9%. It increased from 47.6 to 72.9% in “Archaeological Site of Troy,” from 54.3 to 76.2% in “Diyarbakır Fortress and Hevsel Gardens Cultural Landscape,” and from 57.1 to 78.1% in “Göbekli Tepe.” Statistically significant differences were found between the pre-test and post-test results in all these cultural heritage sites, which clearly shows that the infographic-based activity was effective in increasing the cultural heritage knowledge of young athletes.

The second sub-question investigated whether there was a significant difference between the results of the cultural heritage promotion activity conducted on social media with infographics for sports high school students and the participants’ sports branches (team sports or individual sports). When the independent sample *t*-test data in Table 3 are examined, it can be seen that the pre-test *t*(188) = −0.051/*p* = 0.959 > 0.05; post-test *t*(193) = −0.807/*p* = 0.421 > 0.05. This indicates that there is no statistically significant difference in the total scores of the pre-test and post-test between participants who engage in individual sports and those who engage in team sports.

**Table 3:** Independent sample *t*-test results of the participants’ answers to the questions in the achievement test according to the branches of the participants.

Test	Group	*N*	X	S	*t*	SD	*p*
Pre-test	Individual sport	98	10, 23	0.383	−0.051	188.231	0.959
	Team sports	98	10, 27	0.457			
Post-test	Individual sport	98	14, 11	0.339	−0.807	193.418	0.421
	Team sports	98	14, 51	0.358			

However, differences were observed in some specific cultural heritage areas according to sport branches. For example, a significant difference was found between sports branches in the post-test in the question about “Historic Areas of Istanbul” (*t*(169) = 2.091, *p* = 0.038 < 0.05). Similarly, a significant difference was found between sports branches in the post-test in the question about “Selimiye Mosque and its Social Complex” (*t*(171) = −2.195, *p* = 0.030 < 0.05) and “Diyarbakır Fortress and Hevsel Gardens Cultural Landscape” (*t*(188) = 2.159, *p* = 0.032 < 0.05). These differences suggest that the effect of sports branches on knowledge acquisition in the promotion of certain cultural heritage sites should be examined in more detail.

The third sub-question investigated whether there was a significant difference between the results of the cultural heritage promotion activity conducted on social media with infographics for sports high school students and the region where the participants lived. When the results of the one-way ANOVA test in Table 4 are examined, it is revealed that the mean scores of the groups from the pre-test application showed a significant difference (*F*(6, 203) = 2.237, *p* = 0.041 < 0.05), while the mean scores from the post-test application (*F*(6, 203) = 1.542, *p* = 0.166 > 0.05) did not differ significantly. In order to test the source of the difference in the pre-test result, the Bonferroni test, one of the *Post Hoc* tests, was used since the variances were equal. As a result of the test, it was found that the participants in the Southeastern Anatolia Region differed from the participants in the Aegean Region. This suggests that there were differences in the level of knowledge between the regions at the beginning, but the activity applied eliminated or reduced these differences in the post-test.

**Table 4:** One-way ANOVA test results of the participants’ answers to the questions in the achievement test according to the regions in which the participants live.

Test	Group	KT	SD	KO	*F*	*p*
Pre-test	Between groups	221,324	6	36,887	2.237	0.041
	Within groups	3,347,800	203	16,492		
	Total	3,569,124	209			
Post-test	Between groups	107,867	6	17,978	1.542	0.166
	Within groups	2,366,900	203	11,660		
	Total	2,474,767	209			

Regional differences were observed for some cultural heritage sites. For example, while there were significant differences between regions in the pre-test for the question about “Historic Areas of Istanbul” (*F*(6, 203) = 2,283, *p* = 0.037 < 0.05), “Göreme National Park and the Rock Sites of Cappadocia” (*F*(6, 203) = 5,075, *p* = 0.000 < 0.05), “Hattusha: the Hittite Capital” (*F*(6, 203) = 2,659, *p* = 0.017 < 0.05), “Nemrut Dağ” (*F*(6, 203) = 3.609, *p* = 0.002 < 0.05) and “Xanthos-Letoon” (*F*(6, 203) = 4,096, *p* = 0.001 < 0.05), this difference disappeared in the post-test. Similarly, in the question related to “Ephesus,” significant differences between regions persisted both in the pre-test (*F*(6, 203) = 3.610, *p* = 0.002 < 0.05) and post-test (*F*(6, 203) = 2.989, *p* = 0.008 < 0.05). For the “Pergamon and its Multi-Layered Cultural Landscape,” regional differences were observed both in the pre-test (*F*(6, 203) = 2.660, *p* = 0.017 < 0.05) and post-test (*F*(6, 203) = 2.875, *p* = 0.010 < 0.05). Similarly, in the question related to “Archaeological Site of Troy,” significant differences between regions persisted both in the pre-test (*F*(6, 203) = 2,682, *p* = 0.016 < 0.05) and post-test (*F*(6, 203) = 2,410, *p* = 0.028 < 0.05). For the “Göbekli Tepe,” regional differences were observed both in the pre-test (*F*(6, 203) = 3,346, *p* = 0.004 < 0.05) and post-test (*F*(6, 203) = 2,197, *p* = 0.045 < 0.05). These findings suggest that knowledge of some cultural heritage sites may vary regionally, but the overall activity tends to reduce these differences.

The fourth sub-question investigated whether there was a significant difference between the results of the cultural heritage promotion activity conducted on social media with infographics for sports high school students and the grade of the participants. When the results of the one-way ANOVA test in Table 5 are examined, it is revealed that the mean scores of the groups from the pre-test application (*F*(2, 207) = 0.095, *p* = 0.910 > 0.05) and the mean scores from the post-test application (*F*(2, 207) = 2.072, *p* = 0.128 > 0.05) did not differ significantly. This shows that the grade level of the participants did not change the contribution of the activity to general cultural heritage awareness.

**Table 5:** One-way ANOVA test results of the participants’ answers to the questions in the achievement test according to the participants’ grades.

Test	Group	KT	SD	KO	*F*	*p*
Pre-test	Between groups	3,267	2	1,633	0.095	0.910
	Within groups	3,565,857	207	17,226		
	Total	3,569,124	209			
Post-test	Between groups	48,581	2	24,290	2.072	0.128
	Within groups	2,426,186	207	11,721		
	Total	2,474,767	209			

However, in some specific cultural heritage areas, differences were observed across grade levels. For example, in the question about “Nemrut Dağ,” a significant difference was found between grade levels in the post-test (*F*(2, 207) = 6.998, *p* = 0.001 < 0.05). Similarly, in the question about “City of Safranbolu,” while there was a significant difference between grade levels in the pre-test (*F*(2, 207) = 3.276, *p* = 0.040 < 0.05), this difference disappeared in the post-test. For the question about the “Archaeological Site of Troy,” there was a significant difference between grade levels in the post-test (*F*(2, 207) = 4.689, *p* = 0.010 < 0.05). These findings suggest that knowledge of specific cultural heritage sites may differ by grade level, but in general, the infographic-based intervention contributed to knowledge growth at all grade levels.

## 5 Discussion

In this section, the overall effect of the infographic-based social media activity on the cultural heritage awareness of young athletes and the potential differences of demographic variables (sport branch, region of residence, and grade) on this effect were evaluated.

### 5.1 Evaluation of achievement test results of participants

The percentage values and dependent sample *t*-test results of the achievement tests of the sports high school students who participated in the application show that the activity had a significant effect on the participants’ knowledge of cultural heritage in Turkey, which is protected by UNESCO. When the achievement tests were evaluated in general, it was found that the activity made a statistically significant difference in the test results between the pre-test and post-test (Table 2). This situation reveals that the application was extremely effective in increasing the cultural heritage knowledge of the athletes participating in the activity.

Analyses for each cultural heritage site also support the general finding. Significant and statistically significant increases were observed in the percentage of correct answers from pre-test to post-test for questions related to all cultural heritage sites, such as Great Mosque and Hospital of Divriği, Historic Areas of Istanbul, Göreme National Park and Cappadocia, Hattusha: the Hittite Capital, Nemrut Dağ, Hierapolis-Pamukkale, Archaeological Site of Troy, Diyarbakır Fortress and Hevsel Gardens Cultural Landscape, and Göbekli Tepe. These results confirm that the visual-cognitive advantages of infographics enable abstract and complex cultural heritage concepts to be adapted to the cognitive processing capacities of young people, psychologically optimizing the learning outcome and preparing the ground for permanent knowledge acquisition. By visually simplifying and making information visually appealing, infographics have not only captured the attention of young people but also allowed them to process information with less mental effort. In terms of learning psychology, this is a mechanism that favors more efficient encoding and retrieval of information.

### 5.2 Evaluation of the effect of participants’ sports branches on achievement test results

When the effect of the participants’ sport branches (team sport or individual sport) on cultural heritage awareness was analyzed, no statistically significant difference was found in terms of general achievement test scores (Table 3). This shows that the infographic-based social media activity provided a similar level of knowledge increase for all young athletes regardless of their sport branch.

However, for some specific cultural heritage sites, significant differences were found between sports branches. For example, significant differences were observed between sports branches in the post-test for questions related to Historic Areas of Istanbul, Selimiye Mosque and its Social Complex and Diyarbakır Fortress and Hevsel Gardens Cultural Landscape. These findings suggest that the content or mode of presentation of certain cultural heritage sites may overlap more with the learning styles or interests of athletes in different sports branches. This suggests the potential for customization of content in the design of future cultural heritage promotion activities according to the sport branch of the target audience.

### 5.3 Evaluation of the effect of participants’ region of residence on achievement test results

When the effect of the regions where the participants live on cultural heritage awareness was examined, it was found that there was a significant difference between the regions in the pre-test (Table 4), but this difference was not statistically significant in the post-test. The source of the difference in the pre-test was determined as the difference between the participants in the Southeastern Anatolia Region and the participants in the Aegean Region. This shows that there may be differences in the level of cultural heritage knowledge between the regions at the beginning, but the infographic-based activity applied balanced or reduced these regional differences in the post-test, bringing the knowledge levels closer to each other. This highlights the potential of digital interventions to reduce geographical inequalities.

Nevertheless, regional differences for some specific cultural heritage sites persisted in the post-test (e.g., Ephesus, Pergamon and its Multi-Layered Cultural Landscape, Archaeological Site of Troy, and Göbekli Tepe). This suggests that although some cultural heritage sites are more or less regionally recognized, infographics are effective in increasing overall knowledge, but in some cases regional differences in prior knowledge or interest cannot be completely eliminated.

### 5.4 Evaluation of the effect of participants’ grade on achievement test results

When the effect of the participants’ grade levels (10th, 11th, and 12th grade) on cultural heritage awareness was examined, no statistically significant difference was found in terms of overall achievement test scores in both pre-test and post-test (Table 5). This finding indicates that the infographic-based social media activity provided a similar level of knowledge increase in young athletes at different stages of high school education. This suggests that the material is generally appropriate for the age group and appeals to the cognitive capacities and learning styles of students at different grade levels.

However, significant differences were found between grade levels in the post-test in the questions related to Nemrut Dağ and the Archaeological Site of Troy. In the question related to City of Safranbolu, while there was a significant difference between grade levels in the pre-test, this difference disappeared in the post-test. This suggests that the effect of grade level on knowledge acquisition in the promotion of specific cultural heritage sites should be examined in more detail, but in general, infographics are an effective tool at all grade levels.

## 6 Conclusion and recommendations

This study experimentally investigated the effectiveness of a social media and infographic-based activity in the process of increasing the cultural heritage awareness levels of young athletes. The findings clearly showed that the implemented activity showed a statistically significant increase in the cultural heritage knowledge and awareness levels of young athletes from pre-test to post-test. This result confirms that social media and infographics are highly effective and accessible tools for cultural heritage promotion.

The preservation and transfer of cultural heritage to future generations strengthens the ties of societies with their past, increases the sense of belonging, and supports individual and social psychological well-being. This study supports the idea that cultural heritage is not only a historical value but also a potential resource for an individual’s identity formation and psychological well-being. The role of young people, especially athletes, in the promotion of cultural heritage offers the opportunity to have a profound psychological impact on large audiences through their role-modeling potential and social learning mechanisms. The role of athletes as cultural ambassadors in constructing an identity that reinforces social values expands the scope of the field of sport psychology, drawing attention to their role in social integration and cultural identity construction.

Given the central role of digital media in young people’s lives, the visual-cognitive advantages of infographics likely facilitated improved learning outcomes and may have set the stage for lasting knowledge acquisition. This methodology offers an innovative and psychologically effective approach for the preservation and transmission of cultural heritage to future generations. Moreover, given the bidirectional effects of social media on young people’s identity formation and social interactions, promoting cultural heritage through these platforms has the potential to act as a psychological buffer that strengthens positive aspects while reducing negative risks. This has the potential to increase proactive engagement and social responsibility by encouraging young people to transition from being passive “digital consumers” to becoming active “digital cultural ambassadors” of cultural heritage.

There was no statistically significant difference between the participants’ sports branches, regions of residence and grade levels in terms of general achievement test scores. This suggests that the activity was effective on a wide range of young athletes regardless of demographic differences. However, the observation of significant differences for some specific cultural heritage sites according to demographic variables indicates the potential for future studies to customize content or promotional strategies for more niche audiences.

In the light of the findings of this study, the following recommendations are offered:

- Cultural heritage institutions and ministries of education should disseminate infographics and social media-based cultural heritage promotion activities nationally, taking into account the digital media usage habits of young people. Such interventions can be integrated into existing education systems and contribute to strengthening young people’s cultural identities.
- Sports federations and clubs should invest in programs that support not only the physical performance of athletes but also their cultural and social development. As athletes are important role models in society, training them as cultural heritage ambassadors has the potential to raise awareness among large audiences.
- Future research should examine the long-term effects of infographics and social media activities and explore in-depth changes in young people’s attitudes and behaviors toward cultural heritage. Furthermore, digital promotion methodologies can be developed for different types of cultural heritage (such as intangible heritage).
- Cultural heritage education through digital tools should focus on the development of interactive and gamified content that encourages the active participation of young people. This can accelerate the transformation of young people from passive consumers to active cultural heritage advocates.
- Holistic approaches should be developed that combine cultural heritage promotion with strategies to mitigate the potential negative effects of social media (social comparison, sleep disturbances). This will increase young people’s digital well-being while reinforcing cultural awareness.

Although this study did not aim to directly measure emotional intelligence in young athletes, the potential effects of increasing cultural heritage awareness on psychosocial development should not be overlooked. The literature shows that a strong sense of cultural identity and belonging supports an individual’s skills, such as self-regulation and empathy. Therefore, it is thought that the increased cultural heritage awareness achieved by this study could be a fundamental step in preparing the ground for the emotional intelligence development of young athletes, and that this indirect relationship should be examined in future longitudinal studies.

### 6.1 Limitations of the study

This study has some methodological limitations. First, a single-group pretest-posttest design was used in the research, and no control group was included. This makes it difficult to establish a definitive causal relationship between the increase in participants’ knowledge levels and the intervention itself. For future studies, it is recommended that experimental designs including a control group be used to more clearly demonstrate the effect of the intervention. Second, the sample of the study is limited to students at sports high schools in Turkey. Therefore, the generalizability of the findings to young athletes in different cultural contexts or different educational institutions may be limited.

The exploratory nature of this study and the fact that it was conducted with a specific sample group require caution in interpreting and generalizing the findings. Caution should be exercised when determining the practical implications and magnitude of the effect of the statistically significant differences obtained. Particularly in pilot studies and relatively small samples, it can be difficult to accurately estimate the effect size of the observed results. Therefore, in order to more clearly demonstrate the practical significance of our results, the findings need to be validated in future studies involving larger and more diverse groups of participants.

Furthermore, although the fact that the questions of the test measuring cultural heritage awareness were prepared directly from the infographic content increases content validity, it can be considered a limitation. The observed increase in performance may stem from short-term familiarity with the material presented rather than a deep understanding of the subject. In future research, administering a retention test a certain period after the intervention (e.g., 3–4 weeks later) to measure the persistence and internalization level of the information, or using more interpretation-based questions that require the application of the information to different situations, will increase the validity of the measurement.

## Data Availability

The raw data supporting the conclusions of this article will be made available by the authors, without undue reservation.
